# Supplementary material for: A Comparison of Pre- and Post-Clinical Simulation Anxiety Levels of Undergraduate Medical Students Before and During the COVID-19 Pandemic: A Prospective Cohort Study
Source: Behav Sci (Basel). 2025 Apr 1;15(4):447. doi: 10.3390/bs15040447 (PMC12023953; doi:10.3390/bs15040447)

Supplementary data

Table of contents

Supplementary methods……………………………………………………………3
Strengthening the Reporting of Observational Studies in Epidemiology
Table S1 (STROBE) Statement…………………………………………….…3
Data collection, Missing values, Figure S1 sample size calculation…………..5
Supplementary Results…………………………………………………………..6
- Supplementary Table S2. Posthoc comparison……………………..……6
- Supplementary Table S3. Posthoc comparison……………………….……7
- Supplementary Figure S2. Boxplot…………………………...……………8

Supplementary Methods

Table S1: STROBE Statement—checklist of items that should be included in reports of observational studies

	Item No	Recommendation	Page No
**Title and abstract**	1	(*a*) Indicate the study's design with a commonly used term in the title or the abstract	1
		(*b*) Provide in the abstract an informative and balanced summary of what was done and what was found	3
**Introduction**			
Background/rationale	2	Explain the scientific background and rationale for the investigation being reported	4
Objectives	3	State specific objectives, including any prespecified hypotheses	4
**Methods**			
Study design	4	Present key elements of study design early in the paper	6
Setting	5	Describe the setting, locations, and relevant dates, including periods of recruitment, exposure, follow-up, and data collection	6
Participants	6	(*a*) *Cohort study*—Give the eligibility criteria, and the sources and methods of selection of participants. Describe methods of follow-up<br>*Case-control study*—Give the eligibility criteria, and the sources and methods of case ascertainment and control selection. Give the rationale for the choice of cases and controls<br>*Cross-sectional study*—Give the eligibility criteria, and the sources and methods of selection of participants	6
		(*b*) *Cohort study*—For matched studies, give matching criteria and number of exposed and unexposed<br>*Case-control study*—For matched studies, give matching criteria and the number of controls per case	6
Variables	7	Clearly define all outcomes, exposures, predictors, potential confounders, and effect modifiers. Give diagnostic criteria, if applicable	6
Data sources/ measurement	8*	For each variable of interest, give sources of data and details of methods of assessment (measurement). Describe comparability of assessment methods if there is more than one group	*6*
Bias	9	Describe any efforts to address potential sources of bias	7
Study size	10	Explain how the study size was arrived at	7
Quantitative variables	11	Explain how quantitative variables were handled in the analyses. If applicable, describe which groupings were chosen and why	7
Statistical methods	12	(*a*) Describe all statistical methods, including those used to control for confounding	7
		(*b*) Describe any methods used to examine subgroups and interactions	7
		(*c*) Explain how missing data were addressed	7
		(*d*) *Cohort study*—If applicable, explain how loss to follow-up was addressed<br>*Case-control study*—If applicable, explain how matching of	7

	cases and controls was addressed<br>*Cross-sectional study*—If applicable, describe analytical methods taking account of sampling strategy	
	(e) Describe any sensitivity analyses	7

Continued on next pag

**Results**			
Participants	13*	(a) Report numbers of individuals at each stage of study—eg numbers potentially eligible, examined for eligibility, confirmed eligible, included in the study, completing follow-up, and analysed	9
		(b) Give reasons for non-participation at each stage	9
		(c) Consider use of a flow diagram	9
Descriptive data	14*	(a) Give characteristics of study participants (eg demographic, clinical, social) and information on exposures and potential confounders	9
		(b) Indicate number of participants with missing data for each variable of interest	9
		(c) *Cohort study*—Summarise follow-up time (eg, average and total amount)	9
Outcome data	15*	*Cohort study*—Report numbers of outcome events or summary measures over time	*9*
		Case-control study—Report numbers in each exposure category, or summary measures of exposure	*9*
		Cross-sectional study—Report numbers of outcome events or summary measures	*9*
Main results	16	(*a*) Give unadjusted estimates and, if applicable, confounder-adjusted estimates and their precision (eg, 95% confidence interval). Make clear which confounders were adjusted for and why they were included	9
		(*b*) Report category boundaries when continuous variables were categorized	9
		(*c*) If relevant, consider translating estimates of relative risk into absolute risk for a meaningful time period	9
Other analyses	17	Report other analyses done—eg analyses of subgroups and interactions, and sensitivity analyses	9
**Discussion**			
Key results	18	Summarise key results with reference to study objectives	11-12
Limitations	19	Discuss limitations of the study, taking into account sources of potential bias or imprecision. Discuss both direction and magnitude of any potential bias	12-13
Interpretation	20	Give a cautious overall interpretation of results considering objectives, limitations, multiplicity of analyses, results from similar studies, and other relevant evidence	11,12,13
Generalisability	21	Discuss the generalisability (external validity) of the study results	11,12,13
**Other information**			
Funding	22	Give the source of funding and the role of the funders for the present study and, if applicable, for the original study on which the present article is based	2

*Give information separately for cases and controls in case-control studies and, if applicable, for exposed and unexposed groups in cohort and cross-sectional studies.

Note: An Explanation and Elaboration article discusses each checklist item and gives methodological background and published examples of transparent reporting. The STROBE checklist is best used in conjunction with this article (freely available on the Web sites of PLoS Medicine at http://www.plosmedicine.org/, Annals of Internal Medicine at http://www.annals.org/, and Epidemiology at http://www.epidem.com/). Information on the STROBE Initiative is available at www.strobe-statement.org.

Data collection and missing values
Data was prospectively collected and registered in a database generated with the IBM SPSS Statistics for Apple version 20.0 software. (IBM Corp, Armonk USA). The caseload entry system was test-run to delete unclear or ambiguous items and to verify the adequacy of the data gathering system.
The data present missing values completely at random, therefore, the strategy used (listwise deletion) do not implied biased means, variances or regression weights.

Figure S1: Sample Size

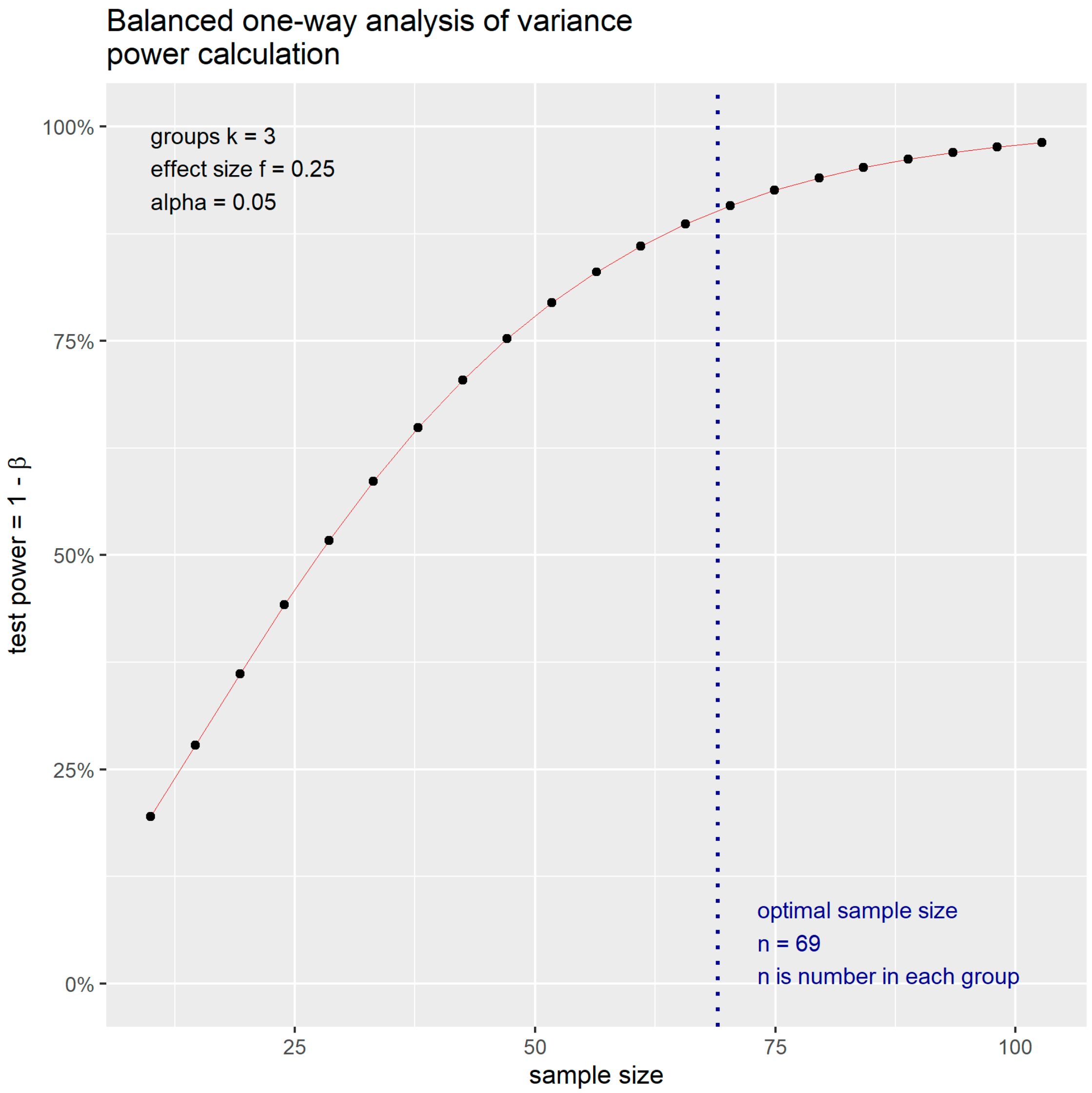

Supplementary table S2: Comparison of baseline characteristics according to scenarios (only pvalue is shown)

Variable	p value
Sex at birth, female	0.830
Age, year	0.008
Body mass index, kg/m2	0.467
Lifestyle habits	
Smoking	0.366
Coffee/tea	0.244
Energy drinks	0.622
Simulation experience, yes	0.521
Leader, yes	0.940
Vital signs	
Oxygen saturation, %	0.069
Perfusion index, %	0.440
SBP, mmHg	0.860
DBP, mmHg	0.073
MBP, mmHg	0.301
Heart rate, beats/min	0.689
Temperature, °C	0.947

Supplementary table S3. Posthoc comparison for those variables with a statistically significant main effect.

a) Initial heart rate

Comparison	pvalue
COVID vs preCOVID	0.676
postCOVID vs preCOVID	0.001
postCOVID vs COVID	0.103

b) Final heart rate

Comparison	pvalue
COVID vs preCOVID	0.881
postCOVID vs preCOVID	0.001
postCOVID vs COVID	0.045

c) Initial diastolic blood pressure

Comparison	pvalue
COVID vs preCOVID	0.015
postCOVID vs preCOVID	0.927
postCOVID vs COVID	0.006

d) Initial mean blood pressure

Comparison	pvalue
COVID vs preCOVID	0.003
postCOVID vs preCOVID	0.309
postCOVID vs COVID	<0.001

e) Initial systolic blood pressure

Comparison	pvalue
COVID vs preCOVID	0.008
postCOVID vs preCOVID	0.027
postCOVID vs COVID	<0.001

f) Final systolic blood pressure

Comparison	pvalue
COVID vs preCOVID	0.678
postCOVID vs preCOVID	0.030
postCOVID vs COVID	0.010

g) Final temperature

Comparison	pvalue
COVID vs preCOVID	0.162
postCOVID vs preCOVID	0.055
postCOVID vs COVID	0.996

Supplementary Figure S2. Boxplot of the statistically significant variables.

a) Initial heart rate

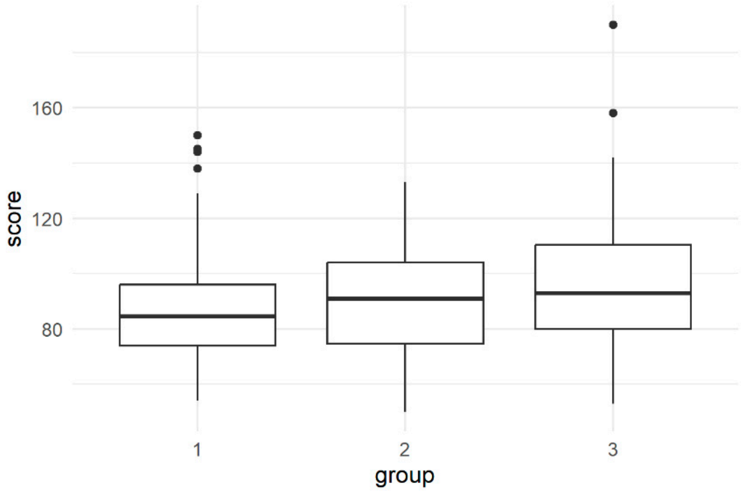

b) Final heart rate

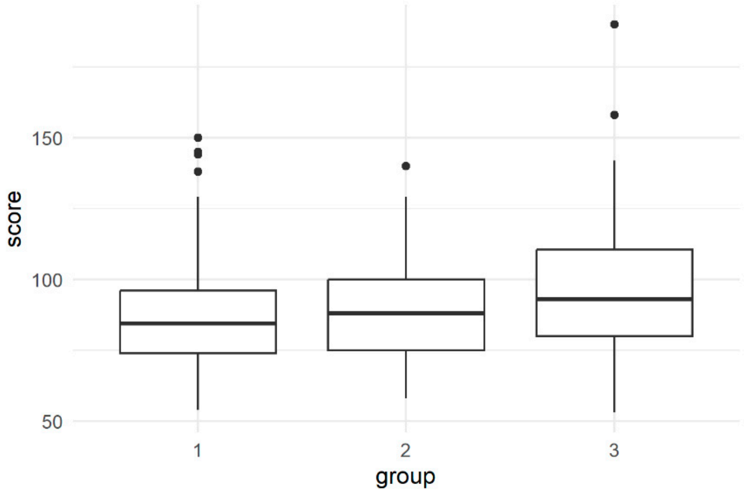

c) Initial diastolic blood pressure

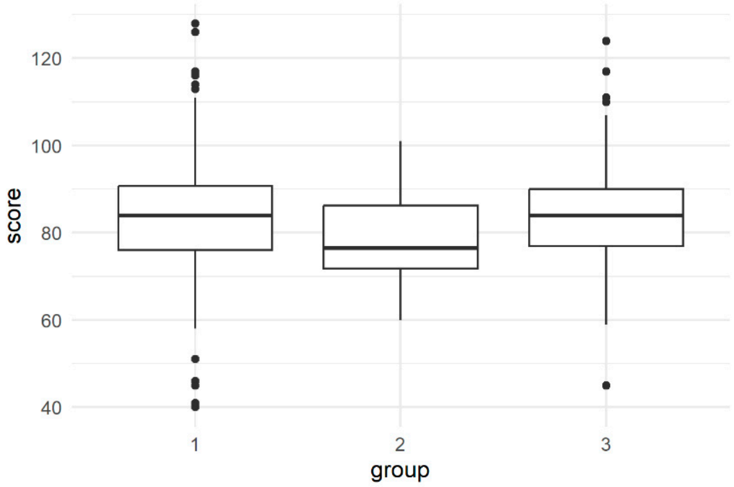

d) Initial mean blood pressure

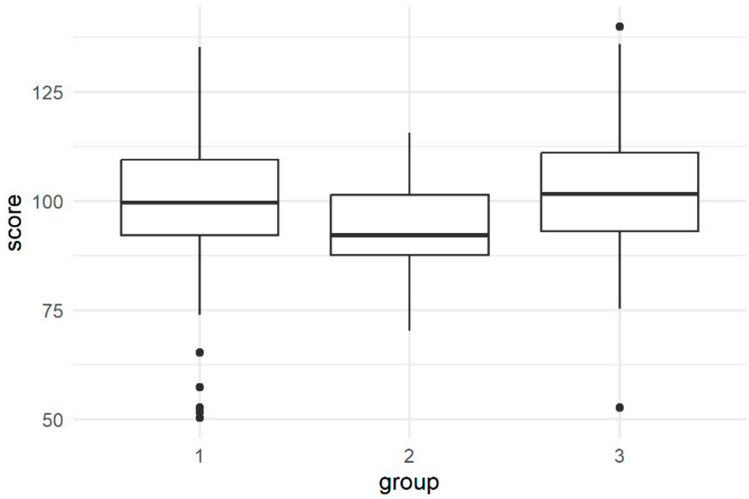

e) Initial systolic blood pressure

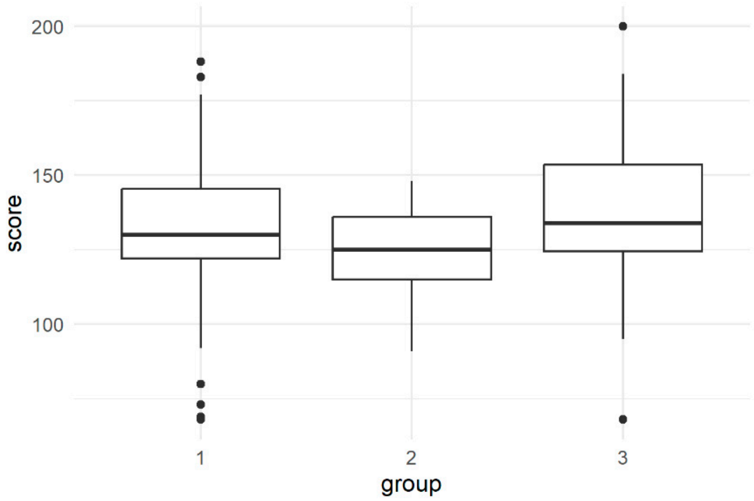

f) Final systolic blood pressure

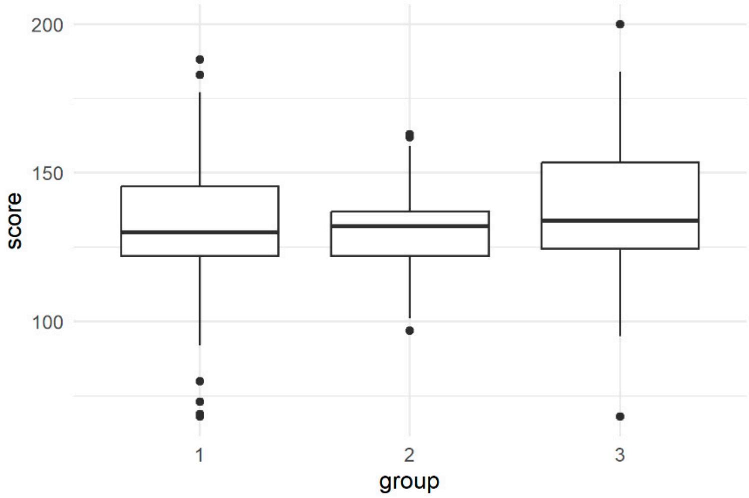

g) Final temperature

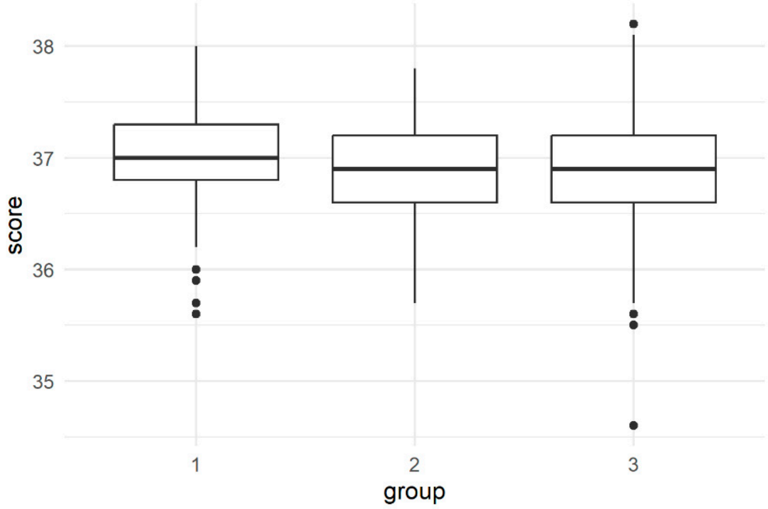

Supplement: Supplementary file 1 [file behavsci-15-00447-s001.zip › behavsci-3502958-supplementary.pdf]
